# Model-Based Prediction of Motor Scores From Sensory Scores in the International Standards for Neurological Classification of Spinal Cord Injury (ISNCSCI): Implications on Motor Levels in Segments Without Clinically Testable Key Muscles

**DOI:** 10.46292/sci25-00012

**Published:** 2025-08-22

**Authors:** Christian Schuld, Viktoria Gavrilova, Laura Heutehaus, Doris Maier, Rainer Abel, Norbert Weidner, EMSCI Study Group, Ruediger Rupp, Steffen Franz, European Multicenter Study about Spinal Cord Injury (EMSCI) Study Group, Rainer Abel, Armin Curt, Axel Hempfing, Yorck-Bernhard Kalke, Jiri Kriz, Doris Maier, Martin Pouw, Norbert Weidner

**Affiliations:** 1 Spinal Cord Injury Center, Heidelberg University Hospital, Heidelberg, Germany; 2 Medical Faculty Heidelberg, Heidelberg University, Heidelberg, Germany; 3 Clinic for Paraplegia, Klinikum Bayreuth GmbH, Bayreuth, Germany; 4 Spinal Cord Injuries, Berufsgenossenschaftliche Unfallklinik Murnau, Murnau, Germany; 5 Department for Spinal Cord Injury, Allgemeine Unfallversicherungsanstalt - Austrian Workers’ Compensation Board, Rehabilitation Center Weisser Hof, Klosterneuburg, Austria; Spinal Cord Injury Center, Klinik Hohe Warte, Bayreuth, German; Spinal Cord Injury Center, Balgrist University Hospital, Zurich, Switzerland; Center for Spinal Cord Injuries, Werner Wicker Hospital, Bad Wildungen, Germany; RKU Universitäts-und Rehabilitationskliniken Ulm, Ulm, Germany; Spinal Cord Unit, Department of Rehabilitation and Sports Medicine, 2nd Faculty of Medicine, Charles University and University Hospital Motol, Prague, Czech Republic; BG Unfallklinik Murnau, Murnau, Germany; Department of Orthopedic Surgery, Radboud University Medical Center, Nijmegen, The Netherlands and Department of Orthopedic Surgery, Sint Maartenskliniek, Nijmegen, The Netherlands; Heidelberg University Hospital, Spinal Cord Injury Center, Heidelberg, Germany

**Keywords:** ISNCSCI, machine learning, motor level, motor score estimation, multiple linear regression, random forest regression, spinal cord injury

## Abstract

**Background:**

In the International Standards for Neurological Classification of Spinal Cord Injury (ISNCSCI), motor levels are inferred from sensory levels for high cervical, thoracic, and low sacral injuries, as key muscles are only assessed in upper and lower extremities. This is known as the “motor follows sensory level” rule.

**Objectives:**

To develop regression models for estimating motor scores from sensory scores in segments without clinically testable key muscles and to validate the consensus-based “motor follows sensory level” approach.

**Methods:**

A total of 6940 ISNCSCI examinations from the European Multicenter Study about Spinal Cord Injury were reviewed. Multiple linear and random forest regression models were trained on scores in clinically testable segments to predict motor from sensory scores of the same spinal segment and side. Models based on ipsilateral light touch or pinprick scores alone, as well as all bilateral sensory scores, were also evaluated. Predicted motor scores were used to recalculate motor levels for the segments without clinically testable key muscles and compared to the true motor levels.

**Results:**

The ipsilateral regression models showed minimal differences (*R*^2^ 0.64-0.65; RMSE 1.34). Normal motor scores were predicted only for normal sensory function; in the linear model, this was captured by the equation: motor score = 0.18 + 1.22 * light touch score + 0.96 * pinprick score. Model-based motor levels were shifted caudally 0.18 segments (linear regression) and 0.32 segments (random forest regression).

**Conclusion:**

As models predict normal motor function only for normal sensory scores, predicted motor levels deviate only marginally, supporting the “motor follows sensory level” rule.

## Introduction

The International Standards for Neurological Classification of Spinal Cord Injury (ISNCSCI) represent the most established neurological assessment for the classification of a spinal cord injury (SCI).^1^ In addition to the severity classified by the American Spinal Injury Association Impairment Scale (AIS) grade, specific levels, that is, motor levels, sensory levels, and the neurological level of injury (NLI), are derived from sensory and motor scores. Sensory scores grade light touch (LT) and pinprick (PP; including sharp-dull discrimination) sensation in 28 dermatomes. Motor scores grade key muscle functions in 5 upper extremity (C5-T1) and 5 lower extremity myotomes (L2-S1). Sensory and motor scores are assessed on both sides of the body.

The motor level is “defined by the lowest key muscle function that has a grade of at least 3, providing the key muscle functions represented by segments above that level are judged to be intact.”^1^ When the motor level is expected to be at a segment without clinically testable key muscle functions (C1C4, T2-L1, S2-S4/5), it is inferred from the sensory level.^1^ This consensus-based principle is commonly known as the “motor follows sensory level” rule.^2^ This leads to a continuous motor level range from C1 to S3, which promises not only better face validity but also sounder statistical properties. This is achieved by preventing accumulation of C5 (or rostral if C5 is scored below 3) motor levels in cases of high cervical injuries, T1 for thoracic injuries, L2 for lumbar injuries (L2 is scored 3 or better with intact motor scores in the upper extremity), and S1 for sacral injuries, which occur due to “the discontinuous nature of the motor examination.”^3^ However, this convention not only increases complexity in classification^2^,^4^,^5^ but also requires caution in clinical trials, as the upper extremity motor score (summing C5-T1) is prone to ceiling effects.^6^,^7^

The ASIA International Standards Committee introduced clarifications for the transition segments C4-C5 and L1-L2, the first segments at and rostral to the most rostral myotome with clinically testable key muscle functions for upper and lower extremity, respectively. They established “virtual” motor scores derived from sensory scores for C4 and L1 in 2009. Only ipsilateral intact PP and LT sensation in C4 (or L1) leads to an assumed normal motor function in C4 (or L1), whereas any other sensory scoring combination leads to an assumed motor score of one.^8^ With this, motor levels at transition segments can be determined consistently.

We have previously generalized the approach of determining “virtual” motor scores at the transition segments C4 and L1 for the EMSCI ISNCSCI calculator^9^ to C2-C4, T2-L1, and S2-S4/5. With the generalization, motor scores become available for all spinal segments, allowing the determination of motor levels in the comparable way as sensory levels covering all segments from C1 to S3 resulting in less classification errors.^9^ This approach lets the motor level follow the sensory level if the sensory level is located in segments without clinically testable key muscles. However, properties, consistency, and validity of this transfer of sensory to motor function at the same spinal segment have not been investigated in a large set of data so far.

The aim of this work was (1) to develop regression models on the basis of a large dataset of motor and sensory scores of segments with testable key muscle functions for estimation of virtual motor scores from sensory scores in segments without clinically testable key muscles, and (2) to use these virtual motor scores to validate the current consensus-based “motor follows sensory level” approach.

## Methods

**Figure 1.**
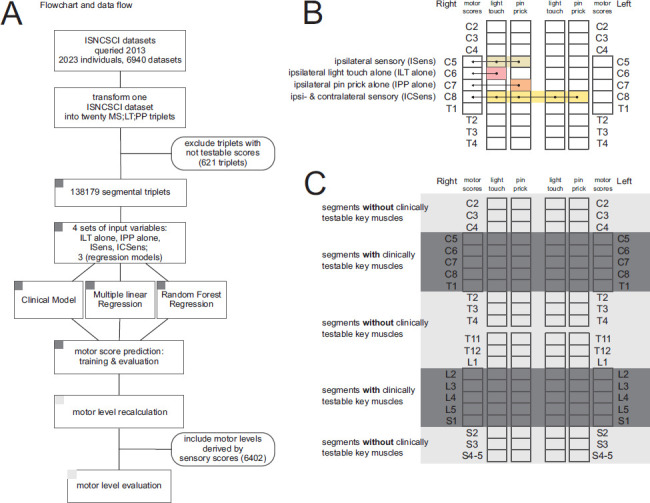
Overview sketch. (**A**) Analysis workflow and data flow. (**B**) The 4 different sets of input variables. (**C**) Myotomes with clinically testable key muscle functions and segments without clinically testable key muscles.

A total of 6940 ISNCSCI exams from 2023 individuals with traumatic or ischemic SCI from the European Multicenter Study about Spinal Cord Injury (EMSCI) database^10^–^12^ were included in the analysis. In a 2-step approach (**Figure 1A**), several regression models and a clinical model for estimation of virtual motor scores were trained from scores of those segments (C5-T1 and L2-S1), where both motor and sensory scores were available (**Figure 1C**). Then, motor levels were determined from combined analysis of virtual motor scores for C2-C4, T2-L1, and S2-S4/5 and true motor scores for C5-T1 and L2-S1, and the deviations from true motor levels were determined. The term *true motor level* refers to the motor level as determined by the current ISNCSCI classification rules, including levels that are inferred from sensory levels.

### First step: Motor score prediction models

This section introduces three models for predicting motor scores: 1 based on clinical knowledge and 2 machine learning models trained on the EMSCI database. Their predictive performance was benchmarked, along with their influence on motor level recalculation in the second step.

#### Clinical model

For the estimation of a virtual motor score from LT and PP scores, virtual motor scores were determined as 5 if LT and PP are normal on the same side, as 0 if LT and PP are absent, and 1 for any other LT and PP scoring.^9^ This clinical model (CLM) represents a generalization of the consensus-based “transition zone” concept,^8^ introduced with the 2009 standard review.

#### Machine learning models

Multiple linear regression (MLR) and random forest regression (RFR), 2 frequently used regression models in neuroscience,^13^ were chosen to estimate motor scores from sensory scores in a data-driven manner. In multiple linear regression, the prediction is based on 2 or more input variables in contrast to ordinary linear regression, where only a single input is used. Linear regression models are characterized by their prediction formulas, whereas random forest models are characterized by their feature importances. Comparable results of both regression models would indicate the interpretability and generalizability of the finding.

The motor score prediction models were trained with the aim of estimating a motor score from sensory scores of the same segment. Training and evaluation are done for all segmental motor and sensory scores of the segments C5-T1 and L2-S1 (**Figure 1C**).

In addition to using ipsilateral segmental LT and PP scores together (feature set: ISens in **Figure 1B**) for the estimation of the motor score of the same side and segment, another 3 sets of sensory scores were used for training distinct models: ipsilateral and contralateral LT and PP (ICSens in **Figure 1B)**, ipsilateral LT (ILT) alone, and ipsilateral PP (IPP) alone (**Figure 1B**). With ILT alone and IPP alone, the question of whether 1 of the 2 sensory examinations might be sufficient for reliably determining virtual motor scores, and in turn motor levels, should be answered. The ILT alone and IPP alone models are based on simple linear regression, not multiple linear regression.

Including additional sensory information from the contralateral side, such as in the ICSens feature set, might boost performance. Specifically, the contralateral PP score may enhance performance, as the contralateral PP score may reflect spinothalamic tract axon decussation.^14^

Coefficient of determination (*R*^2^) and root mean square error (RMSE) were used to evaluate and compare the models in terms of explained variance and measurement error, respectively. Details on the training regimen are presented in the **Appendix**.

### Second step: Motor level recalculation based on predicted motor scores

Motor levels were recalculated on the basis of the virtual motor scores, which were mathematically rounded to the nearest integer, for the segments C2-C4, T2-L1, and S2-S4/5 and the true motor scores from the examined key muscle functions in C5-T1 and L2-S1. Thanks to the availability of motor scores for all spinal segments, the motor level determination is similar in complexity to the sensory level determination process, with the motor level being the most caudal segment graded as 3 or better, provided that all rostral myotomes are graded as intact.

To exclusively evaluate the “motor follows sensory” aspect of the motor level definition, exams were included only where the motor level was determined by inclusion of sensory functions. This includes all motor levels that were inferred by sensory levels and motor levels at the transition zone C4/C5 and L1/L2.

Such motor levels were identified as follows: For each exam, all sensory scores were temporarily replaced with normal scores, and temporary motor levels were recalculated with the EMSCI ISNCSCI calculator. If the temporary motor level differed from the true motor level, it was proven that the true motor level was determined by the inclusion of sensory function.

Python's data science stack was used for data processing (pandas),^15^ regression modeling, statistics (scikit-learn, statsmodels),^16^–^18^ and visualization (matplotlib).^19^ Threshold for significance was set to α = 0.05.

## Results

### Demographics

Basic demographics are reported according to the reporting guidelines specified by the International Datasets consortium.^20^ Age at the time of injury in years is distributed as follows (*n* = 2023 individuals with SCI): 0-15 (1.2%), 16-30 (26.9%), 31-45 (25.2%), 46-60 (22.3%), 61-75 (6.3%), and 76+ (18.1%). NLI and AIS of all ISNCSCI exams (*n *= 6940) are distributed as follows: C1-4 (A: 9.3%, B: 3.0%, C: 4.0%, D: 8.9%), C5-T1 (A: 6.6%, B: 3.6%, C: 3.3%, D: 11.5%), T2-L1 (A: 24.4%, B: 4.6%, C: 5.1%, D: 7.5%), L2-S1 (A: 0.7%, B: 1.1%, C: 1.3%, D: 4.1%), and S2S4/5 (B: <0.1%, D: <0.1%, E: 0.8%).

### Relation between motor and sensory scores in the EMSCI cohort

The most frequent segmental motor and sensory score combination (triplets consisting of a motor score [MS], LT score, and PP score) was normal LT and PP grading with an MS of 5 (intact) (34.4%). This was expected, when including all available 20 triplets. The second most common combination was found to be absent LT, absent PP, and full paralysis (MS = 0) (24.0%). All other scoring combinations are far less prominent, with LT = 1, PP = 0, and MS = 0 (4.5%) and LT = 2, PP = 2, and MS = 4 (4.1%) being the third and fourth most frequent scoring combinations. eTable 1 depicts the confusion matrix of all 138,179 investigated triplets for motor, LT, and PP scores.

### First step: Estimated motor scores from sensory scores

The MLR models and RFR models performed almost equally well and were always better than the CLM (**Table 1**).

**Table 1.** Model evaluation and regression formulas for the clinical model (CLM), multiple linear regression (MLR), and random forest regression (RFR) for 4 different sets of input data

Input variables	Model	*R^2^*	RMSE (motor score)	Prediction formula/feature importances for motor scores
ILT alone	CLM	0.51±0.03	1.58±0.04	5 if ILT=2, 1 if ILT=1 else 0
	MLR	0.61±0.02	1.40±0.03	0.14 + 2.10 ^*^ ILT
	RFR	0.61±0.02	1.40±0.03	ILT 1.00±0.00 ^*1^
IPP alone	CLM	0.45±0.04	1.66±0.04	5 if IPP=2, 1 if IPP=1 else 0
	MLR	0.59±0.02	1.44±0.03	0.61 + 1.92 ^*^ IPP
	RFR	0.59±0.02	1.44±0.03	IPP 1.00±0.00 ^*1^
ISens	CLM	0.50±0.03	1.59±0.04	5 if ILT=2 and IPP=2, 0 if ILT=0 and IPP=0, else 1
	MLR	0.65±0.02	1.34±0.03	0.18 + 1.22 ^*^ ILT + 0.96 ^*^ IPP
	RFR	0.64±0.02	1.34±0.03	ILT 0.72±0.34; IPP 0.28±0.34 ^*1^
ICSens	MLR	0.66±0.02	1.30±0.03	0.14+ 0.76 ^*^ ILT + 0.39 ^*^ CLT + 0.50 ^*^ IPP + 0.56 ^*^ CPP
	RFR	0.66±0.02	1.30±0.03	ILT 0.25±0.19; IPP 0.03±0.04; CLT 0.00±0.00; CPP 0.72±0.18 ^*1^

*Note:* Input data: ILT alone = light touch alone; IPP alone = pinprick alone; ISense = ipsilateral light touch and pinprick; ICSens = ipsilateral and contralateral light touch and pinprice. Sensory scores used in the regression models are denoted as: IPP (ipsilateral PP), ILT (ipsilateral LT), CPP (contralateral PP), and CLT (contralateral LT). The standard deviation of the coefficients for all MLR models are 0.02 except for the IPP/MLR and ICSens/MLR models, which are all 0.01. RMSE = root mean square error.

*1 Feature importances of the random forest regressor.

#### Motor score estimation from ILT and IPP score (ISens feature set)

MLR and RFR models trained from ISens feature set were equal up to the second decimal point in RMSE (1.34) and differ only a hundredth in *R*^2^ (MLR 0.65, RFR 0.64). The CLM derived from the generalized transition zone concept,^8^ which uses the same transfer function as the EMSCI ISNCSCI calculator,^9^ explained approximately 15% less variance with 0.15 motor points more deviation compared to regression model.

The MLR model led to the following motor score prediction formula:



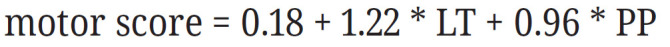




Standard deviations of intercepts and all coefficients (all *P*s < .05) were below or equal to 0.02 for all MLR models. The LT coefficient (1.22) had a stronger linear impact on the motor score estimation than the PP coefficient (0.96). The same relationship of LT having a higher impact was found in the feature importances of the RFR model (LT 0.72 ± 0.34; PP 0.28 ± 0.34).

Predicted motor scores using the 3 models are displayed in **Table 2**. The motor score determined by MLR and RFR only differed in 3 scenarios (LT = 2, PP = 1), (LT = 0, PP = 1), and (LT = 1, PP = 1), but only the latter had any actual meaning on the subsequent motor level recalculation, because it changed the score from 3 (RFR) to 2 (MLR) (**Table 2**).

**Table 2.** Prediction models for the clinical model (CLM), multiple linear regression (MLR), and random forest regression (RFR) using the ISens variable set (ipsilateral light tough light touch score and ipsilateral pinprick score) and the light touch alone (ILT alone) and pinprick alone (IPP alone) feature sets

Occurrence frequencies in ISNCSCI exams, % (N=6940)	Input (examined scores)		Output (predicted scores)		
	Ipsilateral light touch & pinprick (ISens feature set)		CLM	MLR	RFR
	Ipsilateral light touch score	Ipsilateral pinprick score	Expert-based motor scores (MS)	Estimates MS (rounded MS)	
43.45	2	2	5	4.53 (5)	4.53 (5)
3.17	2	1	1	3.57 (4)	3.23 (3)
1.84	2	0	1	2.61 (3)	2.52 (3)
2.64	1	2	1	3.31 (3)	2.64 (3)
13.96	1	1	1	2.35 (2)	2.51 (3)
8.80	1	0	1	1.40 (1)	1.42 (1)
0.08	0	2	1	2.10 (2)	1.66 (2)
0.50	0	1	1	1.14 (1)	1.53 (2)
25.54	0	0	0	0.18 (0)	0.15 (0)
Frequency, %	(Ipsilateral) light touch alone (ILT alone feature set)		CLM	MLR	RFR
	ipsilateral light touch score				
48.47	2		5	4.35 (4)	4.38 (4)
25.40	1		1	2.25 (2)	2.13(2)
26.12	0		0	0.14 (0)	0.18 (0)
Frequency, %	(Ipsilateral) pinprick alone (IPP alone feature set)		CLM	MLR	RFR
	Ipsilateral pinprick score				
46.17	2		5	4.45 (4)	4.44 (4)
17.63	1		1	2.53 (3)	2.57(3)
36.20	0		0	0.61 (1)	0.61 (1)

*Note:* The occurrence frequencies of the light touch and pinprick scores are depicted in the first column “occurrence frequencies.” The estimated motor scores (MS) in parentheses are the mathematically rounded values to the nearest integer used for further analysis of the motor level. Background color coding: 5:  dark gray score lets the motor level (ML) determination process continue (if only 5s are present rostral to this segment) 3-4:  light gray  score stops ML determination process, moves ML one segment caudally 0-2:  white  scores stop motor level determination process If 2 models have the same gray shade, they will determine the same motor level.

Estimated motor scores of 3 or 4 are relevant for motor level determination (given that all rostral segments are graded with examined/virtual motor scores of 5) and are therefore highlighted in the respective tables (**Table 2**, eTable 2), which allows for the rapid comparison and interpretation of the subsequent task of motor level recalculation.

#### Motor score estimation from ILT alone IPP alone (ILT and IPP feature sets)

MLR and RFR model trained on ILT alone and IPP alone show only minor differences (**Table 1**). Notably, the ILT alone models performed slightly better, with *R*^2^ values of 0.61 and RMSE of 1.40 for both MLR and RFR, compared to the IPP alone models (*R*^2^ 0.59, RMSE 1.44).

Neither the MLR nor the RFR model could estimate a motor score of 5, but both predicted values close to 4.4, just below the threshold where rounding would yield a 5 (**Table 2**). Because the ability to estimate an intact motor grade of 5 is essential for determining a motor level, these models were omitted and not further evaluated.

#### Motor score estimation from ipsilateral and contralateral LT and PP scores (ICSens feature set)

By adding contralateral segmental sensory scores into the regression models, only a small increase in performance was achieved compared to the ISens feature set: 1% to 2% more explained variance (MLR 0.65 to 0.66; RFR 0.64 to 0.66), and a slight decrease of RMSE from 1.34 to 1.30 for both regression models (**Table 1**). Contralateral PP (CPP) had a stronger impact (MLR/RFR 0.56/0.72) in both models compared to IPP (MLR/RFR 0.50/0.03) (**Table 1**). In contrast, LT had a stronger impact when ipsilateral (MLR/RFR 0.76/0.25) compared to contralateral (MLR/RFR 0.39/0.00) (**Table 1**).

The results of the prediction models can be provided in the form of lookup tables, because only a limited number of integer sensory scoring are possible, that is, 3 for ILT and IPP, 9 for ISens (**Table 2**), and 81 for ICSens (eTable 2).

### Second step: Motor levels recalculated from estimated motor scores

The differences between the recalculated motor levels, derived from estimated motor scores by the ISens feature set for segments without clinically testable key muscles and the true motor scores from testable segments, and the true ISNCSCI motor levels are presented in the following. Consistent with prior reasoning, motor levels calculated with the estimated motor scores from regression models using the ISens feature set can only match the true motor level or can only be one segment caudal to the true motor level. With MLR estimated motor scores, the recalculated motor level matched the true motor level in 82.34% and was one segment caudal to the true motor level in 17.66% of cases, with an average shift of 0.18 ± 0.38 (median 0, interquartile range [IQR] 0) segments caudally. In comparison, RFR estimated motor scores resulted in a one-segment caudal shift in 31.92% of cases, with an average shift of 0.32 ± 0.47 (median 0, IQR 1) segments caudally.

## Discussion

Motor scores can be estimated from ipsilateral sensory scores of the same segment with basic statistical models explaining a substantial^21^ proportion of the variance (approximately 65%). Both multiple linear regression and random forest regression yield nearly identical performance for ISens (incorporating ipsilateral sensory LT and PP), suggesting that the relationships in the data are predominantly linear. The lack of improvement from a non-linear approach (RFR) indicates that incorporating complex modeling techniques does not provide additional predictive power in this context. This consistency across models supports the stability of the findings and suggests that a simpler, interpretable model may be preferable in practical applications. Logistic regression, which has been successfully used for prediction models in SCI,^22^,^23^ would have been a reasonable alternative, particularly to replicate the clinical model's dichotomous logic of distinguishing between a motor score of 5 and scores below. However, our study pursued a 2-fold aim: (1) to develop a general-purpose prediction model for continuous motor scores based on sensory input, and (2) to explore the clinical assumption that “motor level follows sensory level” in a data-driven manner. As logistic regression inherently models categorical outcomes, it does not align with the general-purpose continuous prediction requirement for our investigation.

Due to the model training in all 20 available key muscles, it is applicable regardless of the injury level. Above-level segments were purposefully included because the objective of the study was to validate the motor level definition, particularly the “motor follow sensory level” rule. Because the motor level marks the transition between normal function above the injury and impairment below, incorporating above- and below-level segments in training was deemed necessary for validating the “motor follows sensory level” rule. LT and PP MLR coefficients were close to 1 for ISens and close to 0.5 for ICSens, indicating that both sensory modalities are needed for motor score estimation.

There is ongoing debate about whether LT or PP is more predictive of motor function. We found LT of the same segment to be consistently more predictive across all models and conditions. This finding aligns with EMSCI's ambulation prediction model,^24^-^27^ which incorporates LT and motor scores but no PP scores in its final analysis.^26^ The higher importance of LT sensation in the prediction models might be partly explained with the subtle scoring difference between LT and PP. To achieve a grade of 1 or 2 in PP, one must be able to reliably distinguish between sharp and dull stimuli.^1^ This additional requirement for a grade higher than 0 is reflected in our data, as grade 0 is far more frequently found (36.2%) in PP compared to LT (26.1%).

Vasquez et al.^28^ reported a comparable relationship that the total LT was on average 10 points higher than total PP. However, they also reported that PP more often determined the sensory level than LT. In our models, LT alone as well as PP alone disqualified for predicting motor scores for motor level determination, because the models were not able to predict a normal motor score when the sensory scores (either LT or PP) were intact (score = 2). However, both models were close to the rounding threshold of 4.5 for normal scores. It should also be acknowledged as a limitation that the ISens models only slightly exceeded this threshold.

Adding contralateral sensory scores of the same segment to the motor score prediction model does not significantly enhance its performance. However, an interesting finding is that CPP has a greater impact than IPP. CPP was even the most important feature in the random forest regressor. This is plausible, as the axons of the spinothalamic tract cells decussate to the opposite side of the spinal cord, usually 1 to 2 spinal segments above the entry segment.^14^ In contrast, ILT was more impactful compared to contralateral LT (CLT).

The clinical motor score estimation model used in this work, generalized from the consensus-based transition zone concept,^8^ performs worse than data-driven regression models in estimating motor scores. This was anticipated, because the clinical model only differentiates between normal function, not normal function and full paralysis, whereas the multiple linear regression and the random forest regression model are designed to predict the full range of motor scores from 0 to 5.

All prediction models agree that a motor score of 5 is exclusively predicted for intact sensory function, which is the grade most relevant for motor level determination. Theoretically, normal motor scores could have been predicted for good, but not normal, sensory scores—for example, normal LT with altered PP—potentially leading to motor levels that shift more than one segment caudally compared to the true motor level. Theoretically, for all ipsilateral models, differences in motor levels can only occur in the rare cases that the regression models predict motor scores of 3 or 4 below the true motor level in segments without clinically testable key muscles, causing the motor level to shift one segment caudally. These theoretical considerations apply to all ipsilateral models, including but not limited to our ISens model.

The adjustment to allow the motor level to shift one segment caudally would potentially add more precision to the motor level, because it is not considered in the current “motor follows sensory level” approach. However, on average, the motor level would shift less than half a segment caudally with the ISens model in the investigated EMSCI cohort. This small shift does not justify changing the “motor level follows the sensory level” rule.

We have also explored recalculated motor levels based on ipsilateral and contralateral models (ICSens). However, this concept of interest is not compatible with the current “motor follows sensory” rule, which relies exclusively on ipsilateral information. Due to this incompatibility, it was not further pursued. Notably, in approximately 5% of cases, the predicted motor level would follow contralateral sensory function.

In general, the ”motor level follows sensory level” is counterintuitive, if the sensory impairments are found between C2 and C4 but the most rostrally tested true motor score of C5 is intact.^2^ An “alternate motor level”--that the motor level defers to a more rostral sensory is not applied when C5 or L2 key muscle strength is intact--has been suggested.^2^,^29^ The alteration is not only more intuitive for clinicians^2^ but is also more predictive of future conversion to motor incomplete status.^29^

In addition to the key muscle functions in the upper and lower extremities, ISNCSCI includes the test for the absence or presence of voluntary anal contraction (VAC) as its lowest tested muscle function. However, VAC only assesses the presence of voluntary function rather than confirming normal function—a requirement for its direct inclusion in the motor level rules. Nonetheless, VAC and potentially also deep anal pressure (DAP) could enhance the training of more advanced future models.

## Conclusion

The models predict normal motor function only for normal sensory function. This outcome is not trivial: Theoretically, the models could have failed to predict normal motor scores altogether or assigned them to other combinations of LT and PP values. That they did not and instead aligned with an expected and clinically plausible pattern supports established clinical intuition. As a result, motor levels estimated from sensory scores of the same segment and side deviate only marginally from the true motor level. Accordingly, this core concept of ISNCSCI's current “motor follows sensory level” rule received data-driven validity.

In future work, more advanced regression models could incorporate not only scores of the same or adjacent segments but also more (ISNCSCI)specific information, for example, VAC and DAP, the zone of partial preservation, the AIS, and so on. These enhanced models have the potential to provide more accurate estimations of the motor score and levels, as well as to estimate not testable or missing scores. This is particularly relevant, as up to 10%^7^ of all ISNCSCI cases contain up to 10 not testable scores. The rostral motor level shifts found with the ipsilateral and contralateral models need to be investigated in future studies.

## Supplementary Material



## APPENDIX

### Supplemental Methods

ISNCSCI exams were pooled over time, merging the 5 EMSCI time frames of <2, 4, 12, 24, and 48 weeks after injury.

Fifty times repeated 80% (training dataset)/20% (evaluation dataset) bootstrapping was used. Training/evaluation dataset splitting was performed on the individual level and not the assessment level, that is, all ISNCSCI exams of one individual with SCI were either entirely used for training or for evaluation but not both.

The random forest regression models are set up to use 10 trees for each forest and a maximal tree depth of 2 for ipsilateral LT alone and ipsilateral PP alone feature sets, 3 for the ipsilateral LT and PP feature set, and 4 for ipsilateral and contralateral LT and PP feature set.

## References

[1] Rupp R, Biering-Sørensen F, Burns SP (2021). International Standards for Neurological Classification of Spinal Cord Injury: Revised 2019. Top Spinal Cord Inj Rehabil.

[2] Franz S, Kirshblum SC, Weidner N, Rupp R, Schuld C, EMSCI study group (2016). Motor levels in high cervical spinal cord injuries: Implications for the International Standards for Neurological Classification of Spinal Cord Injury. J Spinal Cord Med.

[3] Marino RJ, Graves DE (2004). Metric properties of the ASIA motor score: Subscales improve correlation with functional activities. Arch Phys Med Rehabil.

[4] Schuld C, Franz S, van Hedel HJ (2015). International standards for neurological classification of spinal cord injury: Classification skills of clinicians versus computational algorithms. Spinal Cord.

[5] Schuld C, Wiese J, Franz S (2013). Effect of formal training in scaling, scoring and classification of the International Standards for Neurological Classification of Spinal Cord Injury. Spinal Cord.

[6] Buri M, Tanadini LG, Hothorn T, Curt A (2022). Unbiased recursive partitioning enables robust and reliable outcome prediction in acute spinal cord injury. J Neurotrauma.

[7] Weidner N, Abel R, Maier D (2025). Safety and efficacy of intrathecal antibodies to Nogo-A in patients with acute cervical spinal cord injury: A randomised, double-blind, multicentre, placebo-controlled, phase 2b trial. Lancet Neurol.

[8] Waring WP, Biering-Sorensen F, Burns S (2010). 2009 review and revisions of the international standards for the neurological classification of spinal cord injury. J Spinal Cord Med.

[9] Schuld C, Wiese J, Hug A (2012). Computer implementation of the international standards for neurological classification of spinal cord injury for consistent and efficient derivation of its subscores including handling of data from not testable segments. J Neurotrauma.

[10] Bourguignon L, Tong B, Geisler F (2022). International surveillance study in acute spinal cord injury confirms viability of multinational clinical trials. BMC Med.

[11] Curt A, Schwab ME, Dietz V (2004). Providing the clinical basis for new interventional therapies: refined diagnosis and assessment of recovery after spinal cord injury. Spinal Cord.

[12] Scheuren PS, Kramer JLK (2024). Next-gen spinal cord injury clinical trials: Lessons learned and opportunities for future success. EBioMedicine.

[13] Smith PF, Ganesh S, Liu P (2013). A comparison of random forest regression and multiple linear regression for prediction in neuroscience. J Neurosci Method.

[14] Blumenfeld H (2021). Neuroanatomy Through Clinical Cases.

[15] McKinney W (2010). Data Structures for Statistical Computing in Python.

[16] Pedregosa F, Varoquaux G, Gramfort A (2011). Scikitlearn: Machine Learning in P ython. J Machine Learning Res.

[17] Seabold S, Perktold J (2010). Statsmodels: Econometric and Statistical Modeling with Python.

[18] Terpilowski MA (2019). scikit-posthocs: Pairwise multiple comparison tests in Python. J Open Source Software.

[19] Hunter JD (2007). Matplotlib: A 2D graphics environment. Comput Sci Eng.

[20] Biering-Sorensen F, DeVivo MJ, Charlifue S (2017). International Spinal Cord Injury Core Data Set (version 2.0)-including standardization of reporting. Spinal Cord.

[21] Cohen J (1988). Statistical Power Analysis for the Behavioral Sciences.

[22] Belliveau T, Jette AM, Seetharama S (2016). Developing artificial neural network models to predict functioning one year after traumatic spinal cord injury. Arch Phys Med Rehabil.

[23] Rowland T, Ohno-Machado L, Ohrn A (1998). Comparison of multiple prediction models for ambulation following spinal cord injury. Proc AMIA Symp.

[24] Brüningk SC, Bourguignon L, Lukas LP An unsupervised machine learning approach to predict recovery from traumatic spinal cord injury. medRxiv.

[25] Hicks KE, Zhao Y, Fallah N (2017). A simplified clinical prediction rule for prognosticating independent walking after spinal cord injury: A prospective study from a Canadian multicenter spinal cord injury registry. Spine J.

[26] van Middendorp JJ, Hosman AJ, Donders AR (2011). A clinical prediction rule for ambulation outcomes after traumatic spinal cord injury: a longitudinal cohort study. Lancet.

[27] van Silfhout L, Peters AE, Graco M, Schembri R, Nunn AK, Berlowitz DJ (2016). Validation of the Dutch clinical prediction rule for ambulation outcomes in an inpatient setting following traumatic spinal cord injury. Spinal Cord.

[28] Vasquez N, Gall A, Ellaway PH, Craggs MD (2013). Light touch and pin prick disparity in the International Standard for Neurological Classification of Spinal Cord Injury (ISNCSCI). Spinal Cord.

[29] Kirshblum S, Snider B, Botticello A, Benedetto J, Engel-Haber E The role of motor zones of partial preservation in conversion from initially complete to motor incomplete spinal cord injury [online ahead of print February 11, 2025]. Arch Phys Med Rehabil.

