# Supplementary material for: Model-Based Prediction of Motor Scores From Sensory Scores in the International Standards for Neurological Classification of Spinal Cord Injury (ISNCSCI): Implications on Motor Levels in Segments Without Clinically Testable Key Muscles
Source: Top Spinal Cord Inj Rehabil. 2025 Aug 22;31(3):129–39. doi: 10.46292/sci25-00012 (PMC12376152; doi:10.46292/sci25-00012)
Supplement: Supplementary file 1 [file i1945-5763-31-3-129_s01.pdf]

eTable 1. Frequencies of ipsilateral motor scores grouped by ipsilateral light touch and ipsilateral pinprick scores.

**Motor scores (*N*=138,179)**		**0**	**1**	**2**	**3**	**4**	**5**
**Light touch score**	**Pinprick score**						
0	0	23.96%	0.58%	0.30%	0.24%	0.25%	0.21%
	1	0.30%	0.05%	0.02%	0.02%	0.04%	0.06%
	2	0.05%	0.01%	0.00%	0.01%	0.01%	0.01%
1	0	4.48%	1.15%	0.59%	0.68%	1.00%	0.91%
	1	3.78%	1.63%	1.37%	1.75%	2.61%	2.82%
	2	0.35%	0.28%	0.21%	0.39%	0.60%	0.81%
2	0	0.49%	0.22%	0.13%	0.21%	0.34%	0.46%
	1	0.42%	0.26%	0.22%	0.45%	0.71%	1.11%
	2	1.25%	0.87%	0.99%	1.85%	4.15%	34.35%

Note: International Standards for Neurological Classification of Spinal Cord Injury (ISNCSCI) datasets are from the European Multicenter Study about Spinal Cord Injury (EMSCI).

eTable 2. Prediction models for multiple linear regression (MLR) and random forest regression (RFR) for ipsilateral and contralateral (ICSens) feature set

**Frequency in benchmark dataset, %**	**Input (examined sensory scores)**				**Output (predicted motor scores)**	
	**ILT**	**IPP**	**CLT**	**CPP**	**MLR**	**RFR**
40.77	2	2	2	2	4.57	4.58
0.77	2	2	2	1	4.02	3.90
0.32	2	2	2	0	3.46	3.50
0.51	2	2	1	2	4.19	4.49
0.75	2	2	1	1	3.63	3.92
0.25	2	2	1	0	3.07	3.52
0.01	2	2	0	2	3.80	4.49
0.02	2	2	0	1	3.24	3.92
0.08	2	2	0	0	2.68	3.52
0.77	2	1	2	2	4.07	3.74
1.47	2	1	2	1	3.51	3.10
0.24	2	1	2	0	2.95	2.81

(continues)

This supplementary digital material accompanies
Top Spinal Cord Inj Rehabil 2025;31(3):129-139
© 2025 American Spinal Injury Association
www.asia-spinalinjury.org
doi: 10.46292/sci25-00012

eTable 2. Prediction models for multiple linear regression (MLR) and random forest regression (RFR) for ipsilateral and contralateral (ICSens) feature set *(cont.)*

Frequency in benchmark dataset, %	Input (examined sensory scores)				Output (predicted motor scores)	
	ILT	IPP	CLT	CPP	MLR	RFR
0.10	2	1	1	2	3.68	3.63
0.41	2	1	1	1	3.12	3.05
0.15	2	1	1	0	2.56	2.81
0.00	2	1	0	2	3.29	3.63
0.01	2	1	0	1	2.73	3.05
0.03	2	1	0	0	2.18	2.81
0.32	2	0	2	2	3.56	3.74
0.24	2	0	2	1	3.00	3.10
0.83	2	0	2	0	2.44	2.31
0.03	2	0	1	2	3.17	3.63
0.07	2	0	1	1	2.61	3.05
0.29	2	0	1	0	2.05	2.31
0.00	2	0	0	2	2.78	3.63
0.00	2	0	0	1	2.23	3.05
0.06	2	0	0	0	1.67	2.31
0.51	1	2	2	2	3.81	3.29
0.10	1	2	2	1	3.25	2.56
0.03	1	2	2	0	2.69	2.14
1.33	1	2	1	2	3.42	3.29
0.45	1	2	1	1	2.86	2.56
0.17	1	2	1	0	2.31	2.14
0.01	1	2	0	2	3.04	3.08
0.01	1	2	0	1	2.48	2.34
0.03	1	2	0	0	1.92	2.14
0.75	1	1	2	2	3.30	3.32
0.41	1	1	2	1	2.74	2.53
0.07	1	1	2	0	2.18	2.14
0.45	1	1	1	2	2.91	3.21
10.79	1	1	1	1	2.36	2.48
1.08	1	1	1	0	1.80	2.14
0.01	1	1	0	2	2.53	3.21
0.09	1	1	0	1	1.97	2.48
0.31	1	1	0	0	1.41	2.14
0.25	1	0	2	2	2.79	3.32
0.15	1	0	2	1	2.23	2.53
0.29	1	0	2	0	1.67	1.14
0.17	1	0	1	2	2.41	3.21

(continues)

eTable 2. Prediction models for multiple linear regression (MLR) and random forest regression (RFR) for ipsilateral and contralateral (ICSens) feature set *(cont.)*

Frequency in benchmark dataset, %	Input (examined sensory scores)				Output (predicted motor scores)	
	ILT	IPP	CLT	CPP	MLR	RFR
1.08	1	0	1	1	1.85	2.48
5.95	1	0	1	0	1.29	1.14
0.00	1	0	0	2	2.02	3.21
0.04	1	0	0	1	1.46	2.48
0.88	1	0	0	0	0.90	1.14
0.01	0	2	2	2	3.05	1.90
0.00	0	2	2	1	2.49	1.54
0.00	0	2	2	0	1.93	1.67
0.01	0	2	1	2	2.66	1.72
0.01	0	2	1	1	2.10	1.37
0.00	0	2	1	0	1.54	0.78
0.03	0	2	0	2	2.27	1.46
0.00	0	2	0	1	1.71	1.11
0.01	0	2	0	0	1.16	0.84
0.02	0	1	2	2	2.54	1.93
0.01	0	1	2	1	1.98	1.52
0.00	0	1	2	0	1.42	1.67
0.01	0	1	1	2	2.15	1.51
0.09	0	1	1	1	1.59	1.15
0.04	0	1	1	0	1.03	0.78
0.00	0	1	0	2	1.76	1.46
0.23	0	1	0	1	1.21	1.10
0.10	0	1	0	0	0.65	0.84
0.08	0	0	2	2	2.03	1.93
0.03	0	0	2	1	1.47	1.52
0.06	0	0	2	0	0.91	1.67
0.03	0	0	1	2	1.64	1.51
0.31	0	0	1	1	1.08	1.15
0.88	0	0	1	0	0.52	0.78
0.01	0	0	0	2	1.26	1.46
0.10	0	0	0	1	0.70	1.10
24.05	0	0	0	0	0.14	0.11

Note: Prediction model uses ipsilateral light tough appreciation (ILT) and ipsilateral pinprick sensation (IPP) as well as contralateral light touch appreciation (CLT) and contralateral pinprick sensation (CPP). The occurrence frequencies of the (ILT, IPP, CLT, CPP) pairs are depicted in column "frequencies." Background color coding:
5: dark gray score lets the motor level (ML) determination process continue(if only 5s are present rostral to this segment)
3-4: light gray score stops ML determination process, moves ML one segment caudally
0-2: white scores stop ML determination process
